# Safety, Tolerability, and Pharmacokinetic Evaluation of Single and Multiple Doses of the Dipeptidyl Peptidase 1 Inhibitor Brensocatib in Healthy Japanese and White Adults

**DOI:** 10.1002/cpdd.1094

**Published:** 2022-04-11

**Authors:** Helen Usansky, Esther Yoon, Ariel Teper, Jun Zou, Carlos Fernandez

**Affiliations:** ^1^ Insmed Incorporated Bridgewater New Jersey USA; ^2^ Adventist Health Glendale USC Verdugo Hills Hospital, Glendale Memorial Hospital and Health Center Glendale California USA

**Keywords:** bronchiectasis, dipeptidyl peptidase 1, food effect, neutrophil elastase, neutrophil serine protease, pharmacokinetics

## Abstract

Brensocatib, an investigational first‐in‐class, small‐molecule, orally bioavailable, selective, and reversible dipeptidyl peptidase 1 inhibitor that blocks activation of neutrophil serine proteases, is currently under clinical development for the treatment of bronchiectasis and other chronic inflammatory diseases. In a 2‐part phase 1 study, the safety, tolerability, and pharmacokinetics of brensocatib were evaluated in healthy Japanese and White adults. In part A, participants received single and multiple once‐daily doses of brensocatib (10, 25, or 40 mg) or placebo after an overnight fast. In part B, participants received a single oral dose of brensocatib 40 mg on days 1 and 8, with or without food in a crossover fashion. Following a single dose and at steady state, brensocatib exposure was dose dependent, with low to moderate interindividual variability; systemic exposure between Japanese and White participants was similar. Elimination half‐life of brensocatib ranged from 22 to 28 hours, resulting in ≈2‐fold accumulation in maximum plasma concentration and area under the plasma concentration–time curve at steady state. In both ethnic groups, the presence of food slightly delayed brensocatib absorption with time to maximum plasma concentration increased by 0.7 to 1.7 hours, but it had no significant effect on brensocatib exposure (maximum plasma concentration and area under the plasma concentration–time curve). Brensocatib was well tolerated in Japanese and White participants. The most frequently reported treatment‐emergent adverse events were headache and skin exfoliation. No clinically significant vital signs, laboratory abnormalities, or evidence of renal toxicity were observed. The results from this study demonstrate that brensocatib can be administered with or without food and that dose adjustment is unnecessary for Japanese patients when receiving brensocatib treatment.

Bronchiectasis is primarily an inflammatory disease with chronic respiratory infections that result in abnormal and irreversible dilatation of the bronchi. Although bronchiectasis primarily affects women aged ≥65 years, the incidence and prevalence are increasing worldwide with notable geographic variations in etiology and clinical features.^1^, ^2^ Symptoms typically include chronic daily cough, sputum production, fatigue, recurrent respiratory infections, and dyspnea,^1^, ^3^, ^4^ although the frequency and severity of symptoms are highly variable.^5^ Pulmonary exacerbations can be frequent and are independently associated with diminished quality of life, decreased lung function, and increased mortality.^5^, ^6^ Systemic and local airway inflammation and bacterial infection are associated with more frequent and severe exacerbations, and the strongest predictor of future exacerbations is previous exacerbations.^7^ Therefore, targeting both the bacterial colonization and inflammation of bronchiectasis are promising approaches for disrupting the cycle of bronchiectasis pathophysiology.^7^

Use of antibiotics, mucolytics, and airway clearance techniques is the current standard of care for patients with bronchiectasis.^7^ For patients who experience ≥3 exacerbations annually, guidelines recommend long‐term antibiotics; however, antibiotic resistance is a common hurdle in effective bronchiectasis management.^7^ Although use of inhaled corticosteroids to manage inflammation of bronchiectasis is widespread, data regarding the efficacy of their use are limited, and long‐term treatment is not currently recommended.^8^ Thus, treatments that target inflammation in bronchiectasis represent an important unmet need.

Deficits in both the innate and adaptive immune systems, particularly dysfunctional neutrophilic response, contribute to bronchiectasis pathophysiology, anatomic damage to the bronchi, and bacterial infection and colonization.^1^ Sputum neutrophils have been correlated with pulmonary function decline, bacterial colonization, and severe disease and contribute to the functional changes and inflammatory‐associated morbidity seen in patients with bronchiectasis.^4^

Neutrophil elastase, a neutrophil serine protease found within cytoplasmic granules, is associated with exacerbations^7^ and may represent a prospective biomarker for disease severity, a predictor of clinical outcomes, and a therapeutic target.^4^ Neutrophil elastase contributes to extracellular matrix degradation, mucus gland hyperplasia, increased mucus production, ciliary beating rate reduction, and direct damage to the epithelium.^4^ Neutrophil elastase inhibitors for the treatment of bronchiectasis have been investigated in phase 2 studies but have failed to produce a clinically significant benefit.^4^

Activation of neutrophil elastase and other neutrophil serine proteases, including proteinase 3 and cathepsin G, occurs during neutrophil maturation in the bone marrow through the action of dipeptidyl peptidase 1 (DPP1; also known as cathepsin C).^9^, ^10^ Brensocatib (INS1007, formerly AZD7896^11^) is a first‐in‐class, small‐molecule, orally bioavailable, selective, and reversible inhibitor of DPP1 that is under investigation for the treatment of bronchiectasis. Brensocatib is a cytochrome P450 (CYP) 3A4, P‐glycoprotein (P‐gp), and breast cancer resistance protein substrate. It is highly permeable in Caco‐2 monolayers (with an apparent permeability from apical side to basolateral side of 9.03 × 10^–6^ cm/s) and subject to minimal degradation in human intestinal microsomes and hepatocytes (unpublished data). Following oral or intravenous administration of ^14^C‐brensocatib to intact and bile duct–cannulated rats, brensocatib was moderately metabolized, mainly via oxidation, N‐demethylation, hydrolysis, N‐formylation, oxidative dealkylation, and hydration. Brensocatib was the major component in plasma, urine, and feces, but not in bile. In plasma, 5 metabolites were detected, with the most abundant metabolite being ≈3% of total radioactivity exposure and brensocatib as the predominant radioactivity component (>80% of total radioactivity exposure) (unpublished data). In healthy adults, ≈20% of brensocatib was excreted in urine as unchanged (unpublished data). A mass balance and metabolic profiling study is ongoing. Brensocatib is not a significant CYP inhibitor (half maximal inhibitory concentration >30 μM for CYP2B6, CYP2D6, and CYP3A4/5; no effect on CYP1A2, CYP2A6, CYP2C8, CYP2C9, CYP2C19, and CYP2E1) or a significant inducer for CYP1A2, CYP2B6, and CYP3A4. In addition, it is not a significant inhibitor of P‐gp, breast cancer resistance protein, organic anion and cation transporters, organic anion transporting polypeptides, or multidrug and toxin extrusion transporters (half maximal inhibitory concentration >11 μM; unpublished data), suggesting that brensocatib is unlikely to modulate the activity of CYP isozymes and transporters at clinically relevant dose levels. In the presence of CYP3A4 and P‐gp inhibitors of verapamil (240 mg once daily for 5 days) or itraconazole (200 mg twice daily for 6 days) (unpublished data), brensocatib area under the plasma concentration–time curve (AUC) increased by ≈14% to 32% in healthy participants,^12^ indicating a mild effect of CYP3A4 and P‐gp inhibitors on brensocatib exposure. Brensocatib also has been shown to inhibit the activity of neutrophil elastase in blood in an exposure‐dependent manner.^9^

A previously conducted phase 1 study of brensocatib in healthy adult male participants revealed dose‐dependent increases in plasma levels with increasing single doses of brensocatib 5, 15, 35, 50, or 65 mg, with maximum concentrations observed at 0.5 to 1.5 hours after dosing; terminal half‐life was 20 to 26 hours with single dosing and 26 to 34 hours with daily dosing (10, 25, or 40 mg for 21‐28 days); furthermore, single oral brensocatib administration (5‐65 mg) or once‐daily dosing (10‐40 mg) for 28 days also resulted in dose‐dependent brensocatib exposure and inhibitory effects on neutrophil elastase activity in the blood.^9^ In a phase 2 randomized, double‐blind, placebo‐controlled trial that assessed the effect of brensocatib on time to first exacerbation, rate of exacerbations, and sputum neutrophil activity (the WILLOW study; ClinicalTrials.gov identifier: NCT03218917), brensocatib 10 and 25 mg reduced neutrophil elastase and improved clinical outcomes in patients with non–cystic fibrosis bronchiectasis.^3^

In this phase 1 study, we evaluated the safety and pharmacokinetics (PK) of brensocatib in healthy Japanese and White adults following single and multiple oral administration. In addition, food effect on brensocatib PK was assessed.

## Methods

The clinical study protocol, participant information sheet, informed consent form, and relevant study documentation were reviewed and approved by the local independent review board (Aspire IRB, LLC) for the study site, Parexel International Early Phase Clinical Unit (Los Angeles, California). The study was performed in accordance with the ethical principles of the Declaration of Helsinki, the Good Clinical Practice guidelines of the International Council for Harmonisation, and applicable regulatory requirements, including the Health Insurance Portability and Accountability Act. All participants provided written informed consent.

### Study Design

The study was conducted sequentially in 2 parts. Part A was a randomized, double‐blind, placebo‐controlled, dose‐escalation study in which healthy Japanese and White adults were randomized 4:1 to receive single (day 1) and multiple (days 3‐30) oral doses of brensocatib (10, 25, or 40 mg) or placebo (10 Japanese and 10 White participants per dose group). Eligible participants were admitted to the clinical unit for single‐dose evaluation from days −2 to 2 and from days 29 to 31 for steady‐state PK and safety evaluation. Participants self‐administered daily doses after an overnight fast of at least 10 hours on days 4 to 30, with outpatient visits on days 3, 4, 7, 14, 21, 32, 33, and 61 (Figure S1). Brensocatib 10‐ and 25‐mg dosing was conducted in parallel, whereas dosing of the 40‐mg group initiated after at least 12 participants (6 Japanese, 6 White) in the 25‐mg group were evaluated following completion of active treatment. Dosing of the 40‐mg group commenced when the safety of 25 mg was deemed acceptable by both the investigator and the sponsor's (Insmed Incorporated, Bridgewater, New Jersey) medical monitor.

Part B was an open‐label, 2‐period, 2‐sequence crossover study in which 20 healthy Japanese and White participants were randomized 1:1 (10 per group/sequence) to receive a single dose of brensocatib 40 mg under either fasted or fed (high‐fat, high‐calorie breakfast) conditions (Figure S1). Participants were admitted to the clinical unit the day before doses were administered on days 1 and 8 and discharged on days 2 and 9, after collection of the 36‐hour postdose PK sample. The washout period between doses was 7 days. For fed conditions, brensocatib was administered ≈30 minutes after the start of breakfast and within 5 minutes of completion. After dosing, participants were required to remain upright for at least 2 hours and refrain from food and drink for at least 4 hours; water was allowed after 2 hours. For fasted conditions, brensocatib was administered after an overnight fast of at least 10 hours.

### Participants

Participants in both parts A and B were healthy Japanese and White adults (18‐50 years old) with no clinically important abnormalities on a 12‐lead electrocardiogram (ECG). Japanese participants had to meet the following criteria: born in Japan, both parents and all 4 grandparents of Japanese descent, and lived <10 years outside of Japan with no significant change in lifestyle (including diet). White participants were required to verbally identify as White and have 4 White grandparents. Eligible participants were nonsmokers with a body mass index (BMI) between 18.0 and 30.0 kg/m^2^ and weighing 45 to 100 kg. Female participants were required to be postmenopausal, surgically sterile, or actively using highly effective contraception methods. Male participants were required to be nonfertile or actively practicing effective contraceptive methods.

Participants were excluded if they had a clinically significant history or evidence of cardiovascular, respiratory, hepatic, renal, gastrointestinal, endocrine, neurological, immunological, or psychiatric disorder(s) or a serious, chronic, or recurring infection within 6 months before screening. Additional exclusion criteria include any disorder that would interfere with absorption, distribution, metabolism, or excretion of drugs; clinically significant abnormal findings in vital signs; a history of multiple or severe allergies to prescription or nonprescription drugs or foods; and use of over‐the‐counter medications within 7 days before the screening visit (use of 1000 mg/day of paracetamol/acetaminophen or ibuprofen was permitted) or prescription medications within 30 days before the screening visit. Throughout the study, participants were monitored for infections and had regular complete blood counts performed, including differential white blood cell count.

### Pharmacokinetic and Pharmacodynamic Assessments

For part A, blood samples for PK assessments were collected following administration on days 1 and 30 at 0 (predose), 0.5, 0.75, 1, 1.5, 2, 3, 4, 8, 12, 24, 48, and 72 hours after dosing. In addition, predose PK samples were collected on days 4, 7, 14, and 21. PK parameters following single and multiple once‐daily dosing were evaluated using noncompartmental analysis, including the AUC over a dosing interval (AUC_τ_), AUC from time 0 to the last time with quantifiable concentration (AUC_last_), AUC from time 0 to infinite time (AUC_∞_), maximum plasma concentration (C_max_), minimum plasma concentration, time to maximum plasma concentration (t_max_), terminal elimination half‐life (t_1/2_), apparent total drug clearance following oral administration (CL/F), and predose concentration. Plasma concentrations of brensocatib were analyzed using a validated liquid chromatography–tandem mass spectrometry (LC‐MS/MS) with a quantification range of 0.250 to 150 ng/mL.

For part B, blood sampling for PK assessments was performed following single‐dose administration on days 1 and 8 at 0 (predose), 0.25, 0.5, 1, 1.5, 2, 3, 3.5, 4, 6, 8, 10, 12, 24, 36, and 72 hours after dosing. PK parameters included C_max_, t_max_, AUC_last_, AUC_∞_, CL/F, apparent volume of distribution after drug administration, and t_1/2_.

### Safety

Safety was assessed from the signing of informed consent until the end of study and included the following variables: treatment‐emergent adverse events (TEAEs), serious adverse events (SAEs), clinical laboratory tests (clinical chemistry, hematology, coagulation [part A only], and urinalysis), vital signs, 12‐lead ECG, primary Holter monitoring, and physical examinations (including evaluation of teeth and gingiva, and evaluation of palms and soles). Because of the mechanism of action of brensocatib, findings of hyperkeratosis, periodontitis/gingivitis, and other infections were reported as adverse events of special interest (AESIs). Renal function (part A only) was monitored by measuring serum creatinine and blood urea nitrogen, performing routine urinalysis, and measuring neutrophil gelatinase–associated lipocalin and cystatin.

Adverse events (AEs) observed before the first dosing were recorded as medical history. All medical history and AEs were coded using the Medical Dictionary for Regulatory Activities (version 21.1 or higher). A TEAE was defined as an AE that began or worsened in severity after at least 1 dose of study drug was administered. All AEs experienced by a participant were monitored until the event had resolved, any abnormal laboratory values returned to baseline or stabilized, a satisfactory explanation for the changes was identified, or the participant was lost to follow‐up.

### Analytical Methods

The plasma concentrations of brensocatib were analyzed using a validated LC‐MS/MS with dipotassium ethylenediaminetetraacetic acid as an anticoagulant. The chromatographic method consisted of a Pursuit C18 column (Agilent Technologies, Santa Clara, California) and a gradient mobile phase of 0.1% formic acid in acetonitrile/water, with ^13^C6‐brensocatib as an internal standard. Brensocatib and the internal standard were monitored by the precursor and product ions of m/z 421.2→100.1 for brensocatib and m/z 427.2→100.1 for the internal standard using an API 5000 or API 5500 LC‐MS/MS (Sciex, Framingham, Massachusetts). The assay range was 0.250 to 150 ng/mL, with overall precision and accuracy for intra‐ and interassays of 2.2% to 5.9% and −2.7% to 0.3%, respectively.

### Statistical Analyses

All statistical analyses were performed using SAS version 9.3 or higher (SAS Institute Inc., Cary, North Carolina). Analysis of PK data was performed using WinNonlin Professional Software version 8.0 or higher (Certara, LP, Princeton, New Jersey). Continuous data were summarized by race or treatment group (active vs placebo) using descriptive statistics (number of observations, arithmetic mean, standard deviation, minimum, median, and maximum). Categorical data were summarized by race and treatment group in terms of number of participants with data at relevant time points, frequency counts, and percentages. PK concentrations below the limit of quantification (BLQ) at time 0 on days 1 and 30 were considered 0. Postdose BLQ data were considered missing for PK parameter calculation. For mean concentrations and graphic presentation, all BLQ data were imputed as 0.

PK parameters were generated using noncompartmental analysis and were based on the PK population, which included all participants who received at least 1 dose of assigned treatment (safety population) and had evaluable C_max_ and AUC parameters without significant protocol deviations. For part B, the PK population included participants who completed both treatment periods and had sufficient concentration data from both periods to fully derive the PK parameters.

The effect of ethnicity (Japanese vs White) on the PK profile of brensocatib was estimated by determining the geometric mean ratio for each dose‐normalized parameter using an analysis of covariance, with AUC (AUC_τ_, AUC_last_, and AUC_∞_) and C_max_ as dependent variables and race and baseline body weight as independent variables.

The effect of food on the PK of brensocatib was assessed separately for Japanese and White participants using an analysis of variance following transformation of the data, with sequence, treatment group, group and period as fixed effects, and participant (sequence) as random effect; group effect was dropped from the analysis of variance if it was nonsignificant at α = 0.05. Estimates and 90% CIs for the differences between treatment means were computed and converted to ratios by antilog transformation. Results for the geometric mean ratio of treatments (fasted vs fed) were tabulated for C_max_, AUC_last_, and AUC_∞_.

A formal sample size calculation was not performed. The number of participants was chosen on the basis of feasibility, as is typical and standard for a multiple‐ascending‐dose study design and was considered sufficient to meet the study objectives.

## Results

### Participants

A total of 82 participants were enrolled and treated: 62 in part A and 20 in part B. Of the 62 participants who received at least 1 dose of the study drug in part A, 28 (87.5%) Japanese and 30 (100%) White participants completed the study. Four Japanese participants discontinued: 1 treated with brensocatib 40 mg discontinued due to a mild dry throat and mild dyspnea, 1 in the placebo group discontinued due to anemia, and 2 participants withdrew consent (n = 1, placebo; n = 1, brensocatib 25 mg). Age and height were generally consistent between Japanese and White participants across treatment groups (Table 1).

**Table 1:** Demographics and Baseline Clinical Characteristics (Safety Population)

	Brensocatib (N = 49)						Placebo (N = 13)	
	10 mg		25 mg		40 mg			
Part A	Japanese (n = 8)	White (n = 8)	Japanese (n = 9)	White (n = 8)	Japanese (n = 8)	White (n = 8)	Japanese (n = 7)	White (n = 6)
Age, y, mean (SD)	31.5 (6.8)	39.5 (4.4)	35.9 (7.3)	37.4 (10.0)	37.8 (5.7)	37.4 (9.6)	37.6 (7.3)	43.7 (3.9)
Sex, n (%)								
Male	6 (75.0)	2 (25.0)	2 (22.2)	5 (62.5)	5 (62.5)	5 (62.5)	2 (28.6)	3 (50.0)
Female	2 (25.0)	6 (75.0)	7 (77.8)	3 (37.5)	3 (37.5)	3 (37.5)	5 (71.4)	3 (50.0)
Ethnicity, n (%)								
Hispanic	0	4 (50.0)	0	3 (37.5)	0	5 (62.5)	0	3 (50.0)
Non‐Hispanic	8 (100)	4 (50.0)	9 (100)	5 (62.5)	8 (100)	3 (37.5)	7 (100)	3 (50.0)
Height, cm, mean (SD)	165.8 (7.6)	165.3 (8.9)	163.0 (8.2)	173.3 (8.3)	167.6 (9.4)	168.1 (9.1)	164.4 (10.4)	167.0 (14.0)
Body weight, kg, mean (SD)	62.4 (7.7)	69.6 (8.4)	57.8 (4.6)	75.8 (9.3)	67.6 (13.1)	73.9 (14.1)	62.4 (11.3)	73.0 (8.8)
BMI, kg/m^2^, mean (SD)	22.7 (2.2)	25.5 (2.7)	21.9 (2.6)	25.2 (2.3)	24.0 (3.3)	26.0 (3.1)	22.9 (2.7)	26.3 (2.0)

Part B					Japanese (n = 10)	White (n = 10)		
Age, y, mean (SD)					35.0 (10.4)	36.6 (9.6)		
Sex, n (%)								
Male					6 (60.0)	7 (70.0)		
Female					4 (40.0)	3 (30.0)		
Ethnicity, n (%)								
Hispanic					0	3 (30.0)		
Non‐Hispanic					10 (100.0)	7 (70.0)		
Height, cm, mean (SD)					166.4 (7.6)	171.4 (7.6)		
Weight, kg, mean (SD)					63.4 (11.3)	73.9 (10.5)		
BMI, kg/m^2^, mean (SD)					22.8 (2.9)	25.2 (3.6)		

BMI, body mass index.

Of the 20 participants enrolled in part B, 9 (90.0%) Japanese and 10 (100%) White participants completed the study. One Japanese participant in the placebo group discontinued the study per protocol before the second dose due to a decrease from baseline in hemoglobin of >2 g/dL. Age, height, body weight, and BMI were generally consistent between Japanese and White participants (Table 1).

### Brensocatib Single‐ and Multiple‐Dose PK: Part A

**Figure 1:**
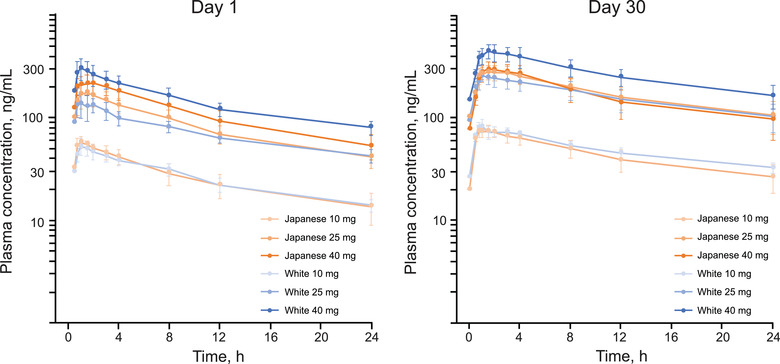
Mean (SD) brensocatib plasma concentrations following a single oral dose (day 1) and once‐daily administration (day 30) (PK population). PK, pharmacokinetics.

The PK population in part A comprised 49 brensocatib‐treated participants (25 Japanese, 24 White). Brensocatib absorption was rapid in all dose groups, with plasma concentrations observed at the first postdose time point (0.5 hour) (Figure 1).

Following single‐dose administration, brensocatib was rapidly absorbed, with a median t_max_ of 1 to 1.5 hours (Table 2). Across brensocatib dose groups, C_max_ and AUCs were approximately dose proportional in both Japanese and White participants. Elimination t_1/2_ ranged from 18.4 to 22.4 hours in Japanese participants and from 21.5 to 24.5 hours in White participants. The intersubject variability in C_max_ and AUCs was low to moderate (12.4% to 48%) across all groups.

**Table 2:** Brensocatib PK Parameters Following Single and Once‐Daily Administration (Part A, PK Population)

	Brensocatib 10 mg		Brensocatib 25 mg		Brensocatib 40 mg	
Parameter	Japanese (n = 8)	White (n = 8)	Japanese (n = 9)	White (n = 8)	Japanese (n = 8)	White (n = 8)
Day 1						
C_max_, ng/mL	64.48 (16.8)	55.85 (26.5)	209.2 (31)	161.1 (38.3)	244.6 (30.1)	328.4 (38.6)
T_max_, h	1.02 (0.75‐2)	1.01 (1‐2)	1.5 (0.75‐8)	1.35 (0.5‐3)	1.5 (0.77‐3.02)	1.27 (0.5‐7.78)
AUC_τ_, ng ‧ h/mL	627.8 (34.2)	619.9 (18.4)	2006 (26.4)	1717 (19.8)	2656 (33.1)	3424 (21.9)
AUC_last_, ng ‧ h/mL	942.7 (43.5)	987.9 (17.1)	3037 (31.4)	2807 (18)	4044 (38.7)	5302 (15.8)
AUC_∞_, ng ‧ h/mL	1026 (46.8)	1136 (19.6)	3388 (35.5)	3205 (17.6)	4585 (45)	5929 (12.9)
t_1/2_, h	18.78 (20.6)	25.68 (34.3)	21.74 (19.7)	24.52 (16)	23.2 (26.7)	22.13 (26.3)
Day 30						
C_max_, ng/mL	80.51 (22.8)	89.46 (17.8)	301.1 (34.1)	265.4 (30.2)	334.3 (30.9)	467.4 (29.5)
T_max_, h	1.26 (0.5‐2)	1.02 (0.77‐3)	1.75 (0.78‐3)	1.02 (0.77‐1.5)	1.03 (0.77‐4)	1.49 (0.78‐3)
C_trough_, ng/mL	15.64 (48.2)	31.65 (52.6)	93.05 (72.3)	83.24 (48.2)	81.86 (101.6)	92.95 (90)
AUC_τ_, ng ‧ h/mL	1051 (36.1)	1185 (14.3)	4166 (39.7)	3873 (38.7)	4008 (44.8)	6466 (30.8)
AUC_last_, ng ‧ h/mL	1729 (44.9)	2103 (19)	6889 (46.2)	6715 (42.1)	6404 (47.9)	10,610 (33.6)
AUC_∞_, ng ‧ h/mL	1985 (49.6)	2542 (25.5)	7894 (49.3)	8160 (45.5)	7112 (48.9)	12,210 (35.9)
t_1/2_, h	22.52 (21.7)	28.14 (21.7)	23.29 (12.1)	28.65 (36.1)	21.91 (14.3)	23.59 (27)
CL/F, L/h	10.78 (39.4)	8.623 (17)	6.889 (39.6)	7.629 (45.9)	11.42 (34.9)	6.801 (34.6)
C_avg_, ng/mL	43.79 (36.1)	49.36 (14.3)	173.6 (39.7)	161.4 (38.7)	167 (44.8)	269.4 (30.8)
C_min_, ng/mL	19.64 (43.2)	26.34 (20.8)	98.84 (52)	93.89 (58.7)	77.49 (68.5)	149.3 (42.3)
RacAUC_τ_	1.668 (14)	1.937 (13.1)	2.097 (14.5)	2.192 (26.6)	1.604 (19.1)	1.949 (32.9)
RacC_max_	1.274 (24)	1.648 (14.4)	1.508 (20.2)	1.75 (30.7)	1.508 (23.9)	1.642 (50.5)

AUC_∞_, area under the plasma concentration–time curve from time 0 to infinity; AUC_τ_, area under the plasma concentration–time curve over the dosing interval; AUC_last_, area under the plasma concentration–time curve from time 0 to the last time with quantifiable concentration; C_max_, maximum plasma concentration; C_min_, minimum plasma concentration; C_trough_, predose concentration; CL/F, apparent total drug clearance following oral administration; CV, coefficient of variation; RacAUC_τ_, accumulation ratio based on AUC_τ_; RacC_max_, accumulation ratio based on C_max_; t_1/2_, terminal elimination half‐life; T_max_, time to maximum plasma concentration.

All values are arithmetic mean (CV%) except T_max_, which is median (range).

Following once‐daily administration, steady‐state C_max_ increased 1.2‐ to 1.7‐fold, and AUCτ increased 1.7‐ to 2.1‐fold. T_max_ (1.0‐1.8 hours) and elimination t_1/2_ (21.7‐27.6 hours) were similar to those after a single dose of brensocatib (Table 2). C_max_ and AUC were approximately dose proportional, with linear slopes of 1.08 and 1.05, respectively, in Japanese participants and 1.17 and 1.20, respectively, in White participants. Across the 3 brensocatib dose groups, mean t_1/2_ was 21.7 to 23.1 hours in Japanese participants and 22.9 to 27.6 hours in White participants (Table 2). Mean steady‐state CL/F was slightly higher in Japanese participants (10.1, 6.4, and 10.7 L/h in the brensocatib 10‐, 25‐, and 40‐mg groups, respectively) compared with White participants (8.5, 7.0, and 6.5 L/h). The dose‐dependent systemic exposure and consistent CL/F and apparent volume of distribution after drug administration across the dose groups demonstrated linear PK of brensocatib.

Following single and multiple dosing, C_max_, AUC_τ_, AUC_last_, and AUC_∞_ were similar between Japanese and White participants treated with either 10 or 25 mg of brensocatib; for those treated with 40 mg, C_max_ and AUCs were lower among Japanese participants (Table 2). Analysis of covariance revealed no differences in C_max_, AUC_τ_, AUC_last_, and AUC_∞_ due to either body weight or BMI. Dose‐normalized C_max_ was 19.1% lower in Japanese participants than in White participants, and dose‐normalized AUCs were ≈25.6% lower in Japanese participants than in White participants (Table 3). Based on body weight– and dose‐normalized AUC_last_, AUC_∞_ after a single dose, and AUC_τ_ at steady state, exposure differences observed between Japanese and White participants were likely due to body weight differences between the ethnic groups (Figure S2).

**Table 3:** Effect of Race on Dose‐Normalized PK Parameters^a^ (Part A, PK Population^b^)

Parameter Single Dose (Day 1)	Japanese n = 25	White n = 24	Ratio of Means (90% CI) for All Participants
C_max,_ (ng/mL)/mg	6.1	6.9	88.1 (74.7‐103.9)
AUC_τ_, (ng ‧ h/mL)/mg	62.5	75.4	82.9 (72.3‐95.1)
AUC_last_, (ng ‧ h/mL)/mg	94.0	118.8	79.2 (67.5‐92.8)
AUC_∞_, (ng ‧ h/mL)/mg	103.9	133.9	77.6 (65.1‐92.6)

Multiple Dosing (Day 30)	n = 23	n = 24	
C_max_, (ng/mL)/mg	8.5	10.6	80.9 (68.6‐95.3)
AUC_τ_, (ng ‧ h/mL)/mg	107.6	144.8	74.4 (60.3‐91.8)
AUC_last_, (ng ‧ h/mL)/mg	172.9	243.8	70.9 (55.8‐90.1)
AUC_∞_, (ng ‧ h/mL)/mg	194.9	285.4	68.3 (52.7‐88.6)

AUC_∞_, area under the plasma concentration–time curve from time 0 to infinity; AUC_τ_, area under the plasma concentration–time curve over the dosing interval; AUC_last_, area under the plasma concentration–time curve from time 0 to the last time with quantifiable concentration; C_max_, maximum plasma concentration; PK, pharmacokinetic.

Data are expressed as geometric least squares mean.

^a^ Analysis based on data pooled from all 3 brensocatib dose groups.

^b^ The PK population for part A included 49 participants (25 Japanese, 24 White) who received brensocatib and had evaluable PK data.

### Brensocatib PK Under Fasted and Fed Conditions: Part B

Following single oral administration of brensocatib 40 mg under fasted conditions, T_max_ was attained at 1.3 hours in both Japanese and White participants (Table 4). C_max_, AUC_last,_ and AUC_∞_ were 338.4, 4820, and 5183 ng · h/mL, respectively, in Japanese participants and 268.9, 5147, and 6201 ng · h/mL, respectively, in White participants. Elimination t_1/2_ was 18.7 and 28.0 hours in Japanese and White participants, respectively, and CL/F was 7.7 and 6.5 L/h in Japanese and White participants, respectively.

**Table 4:** Brensocatib PK Parameters After a Single Oral Dose (40 mg) in Fasted and Fed States (Part B, PK Population^a^)

	Japanese		White	
Parameter	Fasted (n = 10)	Fed (n = 9)	Fasted (n = 10)	Fed (n = 10)
C_max_, ng/mL	351.9 (29.6)	297.4 (25.5)	301.1 (41.1)	302.2 (46.4)
T_max_, h	1.27 (0.48‐3.5)	3 (0.48‐6)	1.25 (0.52‐8)	2 (1‐6)
AUC_last_, ng ‧ h/mL	5012 (31.9)	4980 (33.5)	5480 (34.1)	5473 (30)
AUC_∞_, ng ‧ h/mL	5470 (38.7)	5493 (43.3)	6679 (38.4)	6496 (30.7)
t_1/2_, h	19.28 (27.8)	19.35 (38.3)	28.87 (28)	27.46 (19.7)
CL/F, L/h	8.068 (29)	8.301 (35.5)	7.012 (45.5)	6.868 (38.8)

AUC_∞_, area under the plasma concentration–time curve from time 0 to infinity; AUC_last_, area under the plasma concentration–time curve from time 0 to the last time with quantifiable concentration; CL/F, apparent total drug clearance following oral administration; C_max_, maximum plasma concentration; CV, coefficient of variation; PK, pharmacokinetic; t_1/2_, terminal elimination half‐life; T_max_, time to maximum plasma concentration.

All values are arithmetic mean (CV%) except T_max_, which is median (range).

^a^ The PK population for part B included 20 participants (10 Japanese, 10 White) who received brensocatib and had evaluable PK data.

**Figure 2:**
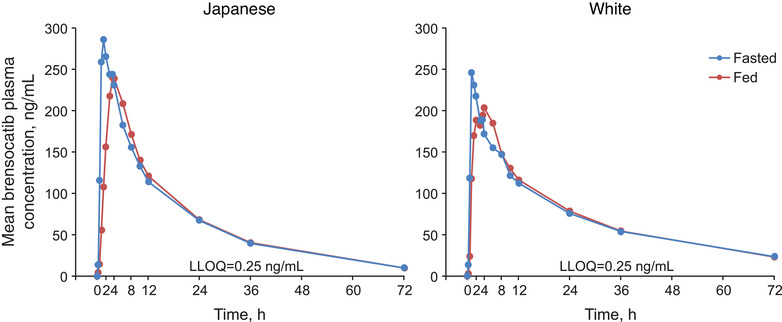
Mean brensocatib plasma concentrations in fasted and fed states (PK population). LLOQ, lower limit of quantification; PK, pharmacokinetics.

The presence of food slightly reduced plasma concentrations among both races (Figure 2) and slightly delayed brensocatib absorption, as the median T_max_ shifted from 1.3 (fasted) to 3.0 (fed) hours in Japanese participants and from 1.3 (fasted) to 2.0 (fed) hours in White participants (Table 4). Mean t_1/2_ was consistent at 18 hours among Japanese participants (18.7 fasted, 18.4 fed) and ranged from 27.0 to 28.0 hours among White participants. CL/F was consistent with or without food in both races. Furthermore, food had no effect on brensocatib 40‐mg exposure (Table 5). Among Japanese participants, C_max_ was 17.6% lower in the fed state. In White participants, C_max_, AUC_last_, and AUC_∞_ were similar under fasted and fed conditions. C_max_ and AUC_last_ were comparable between Japanese and White participants; AUC_∞_ appeared to result from extrapolated AUC from AUC_last_ to infinity.

**Table 5:** Effect of Food Intake on Brensocatib PK (Food Effect Population)

Parameter	Fed	Fasted	GMR, % (90% CI)
Japanese, n=9			
C_max_, ng/mL	293.0	355.8	82.4 (67.9‐99.9)
AUC_last_, ng ‧ h/mL	4840.2	4918.9	98.4 (94.4‐102.5)
AUC_∞_, ng ‧ h/mL	5232.0	5274.0	99.2 (95.7‐102.8)
White, n=10			
C_max_, ng/mL	272.2	268.9	101.2 (81.4‐125.9)
AUC_last_, ng ‧ h/mL	5214.2	5146.6	101.3 (96.8‐106.0)
AUC_∞_, ng ‧ h/mL	6177.7	6201.2	99.6 (94.6‐104.9)

AUC_∞_, area under the plasma concentration–time curve from time 0 to infinity; AUC_last_, area under the plasma concentration–time curve from time 0 to the last time with quantifiable concentration; C_max_, maximum plasma concentration; GMR, geometric mean ratio (fed/fasted); PK, pharmacokinetic.

PK values are expressed as least squares mean.

### Safety

Overall, 65 TEAEs were reported in 37 of 82 participants (45.0%) for parts A and B, with a similar incidence between Japanese (18/42 [42.9%]) and White (19/40 [47.5%]) participants. No SAEs, severe TEAEs, or deaths occurred during the study. No clinically relevant abnormalities were observed, and no treatment‐related or clinically meaningful trends or signals were detected in vital signs, laboratory assessments, Holter monitoring, or ECG data. One Japanese participant in the placebo group experienced a clinically significant abnormal change in hemoglobin (≥2‐g/dL change from baseline) assessed by the investigator as nonserious, mild anemia. All other individual abnormalities were considered to be of no clinical significance. Renal toxicity assessments revealed no signs of acute kidney injury.

In part A, 27 participants (55.1%) treated with brensocatib experienced ≥1 TEAE compared with 4 participants (30.8%) treated with placebo (Supplemental Table 1). The most frequently reported TEAEs (occurring in ≥2 participants in any treatment group) were headache, skin exfoliation, and nasopharyngitis. In part B, 3 Japanese participants (30.0%) and 3 White participants (30.0%) experienced a TEAE (Supplemental Table 1). The most common TEAE was skin exfoliation, reported by 1 Japanese participant (10.0%) and 1 White participant (10.0%). Among Japanese participants, 4 (100%) of the TEAEs reported were under fed conditions and none were reported under fasted conditions; among White participants, 2 (66.7%) of the TEAEs reported were under fed conditions and 1 (33.3%) was reported under fasted conditions.

Physical examinations were normal for all but 6 participants in the study. Three participants in part A and 3 in part B had physical examination findings assessed as AEs; however, all were considered mild to moderate, nonserious events and resolved. In part A, 1 participant experienced abnormal palms and soles, 1 experienced abnormal gingiva and teeth, and 1 experienced abnormal skin. In part B, 2 participants experienced abnormal skin, and 1 experienced abnormal palms and soles.

## Discussion

Because race may be associated with variable drug response,^13^, ^14^ the safety, tolerability, and PK of single and once‐daily oral doses of brensocatib (10, 25, and 40 mg) were evaluated in Japanese and White participants in this phase 1 study. In addition, the food effect on brensocatib systemic exposure was assessed in Japanese and White participants with a single oral administration of brensocatib 40 mg immediately following a high‐fat, high‐calorie breakfast.

Following oral brensocatib administration, linear and predictable PK were observed in healthy Japanese and White participants, characterized by rapid oral absorption; dose‐proportional exposure; consistent t_1/2_, CL/F, and volume of distribution across doses; and ≈2‐fold accumulation at steady state. Based on similar dispositional parameters between a single dose and multiple doses at steady state, brensocatib clearance appeared to be time invariant. When brensocatib was administered with food, its oral absorption was slightly delayed but overall exposure was unchanged, indicating a minimal food effect on oral absorption of brensocatib. Based on these results, brensocatib can be administered with or without food.

Brensocatib exposure following 10‐ and 25‐mg dosing was generally comparable between Japanese and White participants. Following 40‐mg dosing, brensocatib exposure in White participants was higher than that in Japanese participants, leading to an overall moderately higher exposure (dose‐normalized C_max_ and AUC) in White participants. Since the body weight of Japanese participants was generally lower than that of White participants, exposure differences were evaluated using pooled data from parts A and B. The body weight– and dose‐normalized AUC_last_ after single and repeated dosing, AUC_∞_ after a single dose, and AUCτ at steady state were nearly comparable between Japanese and White participants, with a mean (median) ng • h/mL of 2.47 (2.04) vs 2.44 (1.96) for AUC_last_ and 2.07 (1.88) vs 2.01 (1.93) for AUC_∞_/AUCτ, indicating that exposure differences between the ethnic groups were due to body weight differences. From a population PK analysis, race was not identified as a significant PK covariate. Preclinical data showed that brensocatib is highly absorbed, minimally metabolized in the liver, and not a significant CYP450 or transporter substrate or modulator (internal unpublished data); therefore, PK polymorphism is not expected for the drug. Investigation of the effect of food on brensocatib PK revealed a negligible food effect in White participants.

Single and multiple administration of brensocatib (10, 25, and 40 mg) for 28 days was well tolerated in healthy Japanese and White participants, with a safety profile that was similar under both fed and fasted conditions. Of 62 participants in part A, only 2 (3.2%; both Japanese) were withdrawn from the study due to the occurrence of mild AEs (mild dyspnea and mild dry throat, n = 1; mild anemia, n = 1). In part B, 1 participant (5.0%) was withdrawn due to a clinically abnormal laboratory finding (low hemoglobin [7.3 g/dL]). Overall, the most frequently reported TEAEs included headache, skin exfoliation, dry skin, upper abdominal pain, and decreased appetite. No deaths or life‐threatening SAEs were reported. Food had no effect on the safety profile of brensocatib.

Because of the mechanism of action of brensocatib (DPP1 inhibition), hyperkeratosis, periodontitis/gingivitis, and other (nonpulmonary) infections were monitored as AESIs. Monitoring for these AESIs was informed by the clinical presentation of a rare, autosomal recessive condition, Papillon‐Lefèvre syndrome, in which the genetic absence of DPP1 results in diffuse palmoplantar keratoderma and severe periodontitis with premature tooth loss of both deciduous and permanent teeth.^15^ None of these AESIs were evident in this study; the few participants with physical examination findings of abnormal skin, gingiva, or palms and soles were not withdrawn, and all events resolved.

The PK results from the current study are generally in agreement with the results from a first‐in‐human, randomized, placebo‐controlled, dose‐escalation study conducted in healthy male participants treated with a brensocatib oral solution.^9^ After administration of single‐oral‐dose solutions of brensocatib 5, 15, 35, 50, and 65 mg or once daily at 10, 25, and 40 mg under fasting conditions, AUC and C_max_ increased in a generally dose‐proportional manner, and the steady‐state AUC and C_max_ were comparable to those from the current study. Administration of a single 35‐mg dose of brensocatib following a high‐fat breakfast resulted in delayed absorption (T_max_ of 3.3 hours) and decreased C_max_ and AUC by 36% and 9%, respectively. Like the safety results presented in the current study, both single and multiple ascending doses of brensocatib were well tolerated.

## Conclusions

The PK data from this phase 1 study indicate that brensocatib exposure was generally comparable between Japanese and White participants, with linear and predictable PK characteristics. Therefore, no dose adjustment for brensocatib is necessary in Japanese patients. Based on the minimal food effect on brensocatib oral absorption, brensocatib can be administered with or without food. Brensocatib was well tolerated in healthy participants, and the safety profile was similar under fasted and fed conditions. No serious TEAEs occurred, and TEAEs of interest (ie, skin and dental events) were mild or moderate. The PK and safety data from this study support further clinical development of brensocatib.

## Conflicts of Interest

H.U., A.T., J.Z., and C.F. are employees of Insmed Incorporated, Bridgewater, New Jersey. E.Y. has nothing to disclose.

## Funding

The phase 1 study and analyses presented in this publication were funded by Insmed Incorporated, Bridgewater, NJ. The Open Access fee for the published article also was supported by Insmed Incorporated.

## Supporting information

Supporting Information
