# Supplementary material for: Safety, Tolerability, and Pharmacokinetic Evaluation of Single and Multiple Doses of the Dipeptidyl Peptidase 1 Inhibitor Brensocatib in Healthy Japanese and White Adults
Source: Clin Pharmacol Drug Dev. 2022 Apr 11;11(7):832–42. doi: 10.1002/cpdd.1094 (PMC9322451; doi:10.1002/cpdd.1094)
Supplement: Supplementary file 1 — Supporting Information [file CPDD-11-832-s001.docx]

**Supplemental Information**

**Safety, Tolerability, Pharmacokinetic, and Pharmacodynamic Evaluation of Single and Multiple Doses of the Dipeptidyl Peptidase 1 (DPP1) Inhibitor Brensocatib
in Healthy Japanese and White Adults**

Helen Usansky, Esther Yoon, Ariel Teper, Jun Zou, Carlos Fernandez

**Table of Contents**

[**Figure S1.** Study Design 2](#_Toc92814785)

[**Figure S2.** Body Weight– and Dose-Normalized Brensocatib AUC in Japanese and White Participants Under Fasted Conditions 3](#_Toc92814786)

[**Supplemental Table 1.** Summary of Safety (Safety Population) 4](#_Toc92814787)

# **Figure S1.** Study Design


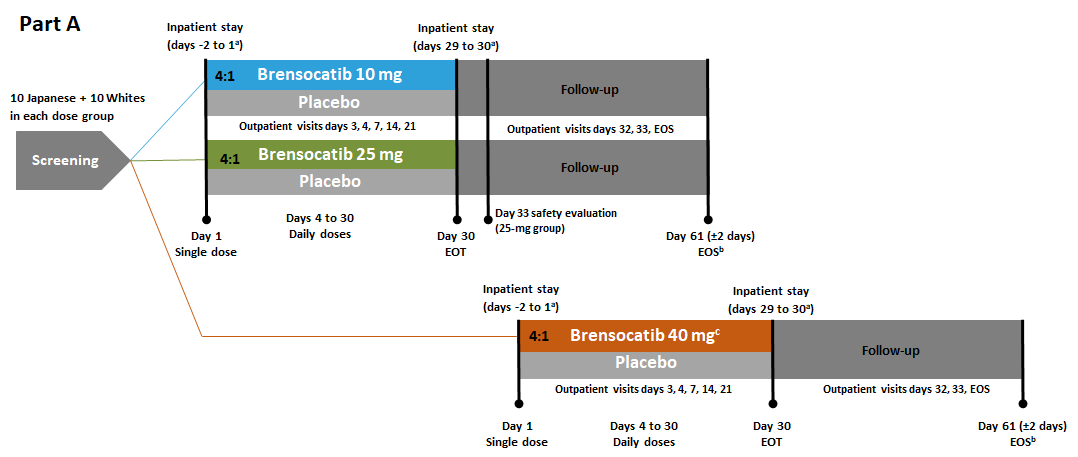


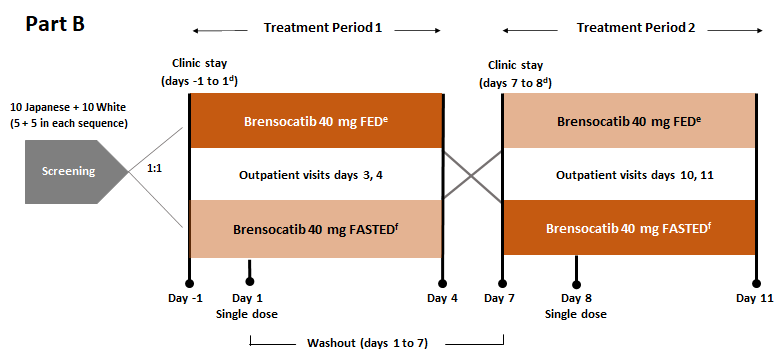
EOS, end of study; EOT, end of treatment; PK, pharmacokinetic.
^a^ Discharge from the clinic occurred on the morning of days 2 and 31, following a review of safety data.
^b^ EOS was defined as completion of the final follow-up visit; for participants who withdrew from the study prematurely, EOS was the time of the last data collection.
^c^ Dosing for the brensocatib 40-mg group was initiated following evaluation of safety and tolerability data for the 25-mg group through day 33 (ie, treatment for the brensocatib 10-mg and 25-mg groups was conducted in parallel, whereas the 40-mg group was initiated after ≥12 participants in the 25-mg group [6 Japanese, 6 White] were evaluated following completion of active treatment).
^d^ Discharge from the clinic on days 2 and 9 after collection of the 36-hour postdose PK sample.
^e^ Brensocatib 40 mg administered approximately 30 minutes after the start of a high-fat, high-calorie breakfast and within 5 minutes of meal completion.
^f^ Brensocatib 40 mg administered after a 10-hour fast; participants continued to fast for ≥4 hours after dosing.

# **Figure S2.** Body Weight– and Dose-Normalized Brensocatib AUC in Japanese and White Participants Under Fasted Conditions


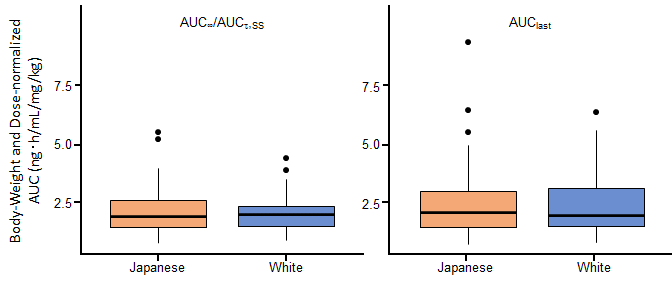


AUC_last_ data were from participants who received a single dose of brensocatib (day 1 for part A and day 1 or 8 under fasted state for part B); AUC_∞_ data were collected after a single dose under fasted state from parts A and B; AUC_τ,ss_ data were from day 30 (part A), assuming that AUC_τ,ss_ equalled AUC_∞_ after a single dose. The boxes represent the interquartile range (IQR) of 25th and 75th percentiles, with the median value shown within the boxes. The whiskers on either side of the IQR represent the lowest and highest data quartiles (≈5% and 95% percentiles), with the end dots representing outlier values. AUC_∞_, AUC from time 0 to infinite; AUC_τ,ss_, area under the plasma concentration-time curve over the dosing interval at steady state; AUC_last_, area under the plasma concentration-time curve from time 0 to the last time point with quantifiable concentration.

# **Supplemental Table 1.** Summary of Safety (Safety Population)

| **Part A, n (%)** | **Brensocatib 10 mg** | | **Brensocatib 25 mg** | | **Brensocatib 40 mg** | | **Pooled brensocatib (N=49)** | **Pooled**  **placebo (N=13)** |
| --- | --- | --- | --- | --- | --- | --- | --- | --- |
|  | **Japanese**  **(n=8)** | **White**  **(n=8)** | **Japanese**  **(n=9)** | **White**  **(n=8)** | **Japanese**  **(n=8)** | **White**  **(n=8)** |  |  |
| Any TEAE^a^ | 3 (37.5) | 6 (75.0) | 5 (55.6) | 5 (62.5) | 3 (37.5) | 5 (62.5) | 27 (55.1) | 4 (30.8) |
| SAE | 0 | 0 | 0 | 0 | 0 | 0 | 0 | 0 |
| Severe TEAE | 0 | 0 | 0 | 0 | 0 | 0 | 0 | 0 |
| TEAE leading to discontinuation | 0 | 0 | 0 | 0 | 1 (12.5) | 0 | 1 (2.0) | 1 (7.7) |
| TEAE leading to death | 0 | 0 | 0 | 0 | 0 | 0 | 0 | 0 |
| **TEAEs in ≥2 participants in any treatment group** | | | | | | | | |
| Headache | 0 | 1 (12.5) | 3 (33.3) | 1 (12.5) | 0 | 2 (25.0) | 7 (14.3) | 2 (15.4) |
| Skin exfoliation | 1 (12.5) | 0 | 2 (22.2) | 1 (12.5) | 0 | 1 (12.5) | 5 (10.2) | 0 |
| Nasopharyngitis | 0 | 2 (25.0) | 0 | 0 | 0 | 0 | 2 (4.1) | 1 (7.7) |
| **Part B, n (%)** | **Japanese** |  |  |  | **White** |  |  |  |
|  | **Fed (n=5)** | **Fasted (n=5)** | **Total (N=10)** |  | **Fed (n=5)** | **Fasted (n=5)** | **Total (N=10)** |  |
| Any TEAE^a^ | 3 (60.0) | 0 | 3 (30.0) |  | 2 (40.0) | 1 (20.0) | 3 (30.0) |  |
| Serious TEAE | 0 | 0 | 0 |  | 0 | 0 | 0 |  |
| Severe TEAE | 0 | 0 | 0 |  | 0 | 0 | 0 |  |
| TEAE leading to discontinuation | 0 | 0 | 0 |  | 0 | 0 | 0 |  |
| TEAE leading to death | 0 | 0 | 0 |  | 0 | 0 | 0 |  |
| **TEAEs by preferred term** | | | | | | | | |
|  | n=9 | n=10 | N=10 |  | n=10 | n=10 | N=10 |  |
| Abdominal pain upper | 1 (11.1) | 0 | 1 (10.0) |  | 0 | 0 | 0 |  |
| Decreased appetite | 1 (11.1) | 0 | 1 (10.0) |  | 0 | 0 | 0 |  |
| Headache | 1 (11.1) | 0 | 1 (10.0) |  | 0 | 0 | 0 |  |
| Dry skin | 0 | 0 | 0 |  | 1 (10.0) | 0 | 1 (10.0) |  |
| Skin exfoliation | 1 (11.1) | 0 | 1 (10.0) |  | 1 (10.0) | 0 | 1 (10.0) |  |
| Skin lesion | 0 | 0 | 0 |  | 0 | 1 (10.0) | 1 (10.0) |  |

SAE, serious adverse event; TEAE, treatment-emergent adverse event.
^a^ TEAE was defined as an adverse event that began or worsened in severity after ≥1 dose of study drug was administered.
